# Molecular and Macromolecular Changes in Bottle-Aged White Wines Reflect Oxidative Evolution–Impact of Must Clarification and Bottle Closure

**DOI:** 10.3389/fchem.2018.00095

**Published:** 2018-04-06

**Authors:** Christian Coelho, Perrine Julien, Maria Nikolantonaki, Laurence Noret, Mathilde Magne, Jordi Ballester, Régis D. Gougeon

**Affiliations:** ^1^UMR A 02.102 PAM Laboratoire PCAV AgroSup Dijon, Université de Bourgogne, Institut Universitaire de la Vigne et du vin Jules Guyot, Dijon, France; ^2^UMR UB/INRA/Centre National de la Recherche Scientifique, Centre des Sciences du Goût et de l'Alimentation, Equipe Culture, Expertise et Perception, Dijon, France

**Keywords:** white wine, colloidal content, proteomic, must clarification, sensory analysis, oxidation

## Abstract

Chardonnay wines from Burgundy, obtained from musts with three levels of clarification (Low, Medium and High) during two consecutive vintages (2009 and 2010) and for two kinds of closures (screw caps and synthetic coextruded closures) were analyzed chemically and sensorially. Three bottles per turbidity level were opened in 2015 in order to assess the intensity of the reductive and/or oxidative aromas (REDOX sensory scores) by a trained sensory panel. The chemical analyses consisted in polyphenols and colloids quantification, followed by a proteomic characterization. For the two vintages, the REDOX sensory scores appeared to be driven both by the type of closure and to a lesser extent by the level of must clarification. Vintages and must racking prefermentative operations were also distinguished by chemical analyses. All white wines from the lowest must turbidity had the lowest REDOX sensory scores. Such wines exhibited lower concentrations in tyrosol and grape reaction product and higher concentrations in colloids with relatively low molecular weights. Among these macromolecules, grape proteins were also quantified, two of them exhibiting concentrations in bottled wines, which were statistically correlated to oxidative evolution in white wines.

## Introduction

Winemaking pre-fermentative operations influence the progress of fermentations and the final quality of wine (Groat and Ough, 1978; Alexandre et al., 1994; Gawel et al., 2014). One of them, which is typical to white winemaking, consists in racking the must before alcoholic fermentation starts. Such practice enables to control must turbidity, a key parameter in the elaboration of white wine. In practice, the winemaker can easily use a turbidimeter in order to control the level of must clarification. Values of turbidity are expressed in NTU (Nephelometric Turbidity Unit). Generally low values for must turbidity, comprised between 50 and 150 NTU, are used for white winemaking in order to prevent reductive aromas and facilitating alcoholic fermentation (Groat and Ough, 1978; Ollivier et al., 1987; Guilloux-Benatier and Feuillat, 1993; Alexandre et al., 1994; Guilloux-Benatier et al., 1995; Gerbaux and Meurgues, 1996; Boivin et al., 1998). However this decision may be difficult to be taken by the winemaker since it depends on external parameters like the vintage, the harvest quality, the terroir, or the desired wine style (Ribérau-Gayon et al., 1975; Groat and Ough, 1978; Williams et al., 1978; Liu et al., 1987).

In any case, must clarification has an impact on the juice content and subsequently on wine composition and sensory attributes. Most of previous studies focused on the description of volatile compounds. Must racking has a positive effect on the fermentative aromas on white wines by decreasing the concentrations of C6 alcohols, coming from the withdrawal of lipidic precursors from the solid deposits, thus reducing the herbaceous characteristics of wines (Ferreira et al., 1995). Additionally, higher alcohols produced during the Ehrlich pathway, presenting heavy olfactive notes, tend also to decrease with an increasing level of must clarification (Liu et al., 1987; Ancín et al., 1996; Casalta et al., 2012). Finally the reductive notes brought by heavy sulfur compounds and the volatile thiol fraction have been shown to diminish with decreasing values of must turbidity (Lavigne, 1996; Lavigne and Dubourdieu, 1997). In some cases, the floral and fruity character is increased in wines elaborated with clarified juices (Williams et al., 1978; Liu et al., 1987).

In addition to the effect on yeast metabolism, must turbidity also affects yeast macromolecules release. Even if no difference is observed in the total protein content (Ayestaran et al., 1995), more exocellular and parietal polysaccharidic yeast compounds are liberated during alcoholic fermentation of clarified musts (Guilloux-Benatier et al., 1995; Boivin et al., 1998). Mannoproteins have been shown to increase the mouthfeel sensation of fullness in wines (Vidal et al., 2004). Among the colloidal diversity of wine composition, grape acidic carbohydrates are also linked to the sensorial qualities of wines. Studies carried on synthetic wines show that proteoglycans seem to lower bitterness perception while acidic polysaccharides reduce astringency (Riou et al., 2002; Vidal et al., 2004). Nevertheless, due to their colloidal behavior and interaction with polyphenols in real wines, further investigation is required to better assess the role of macromolecules in wine sensory perceptions (Ayestaran et al., 2004). White wine mouthfeel and taste perception was recently shown to be more correlated to the polyphenolic content than to the polysaccharidic content (Gawel et al., 2014).

White wines elaborated with different levels of must turbidity have also been studied for their browning rate upon oxidation and no clear evidence emerged (Williams et al., 1978). Only pectinase treated must with the lowest turbidity led to wines with a lowest browning rate, indicating the possible link of the osidic fraction on wine browning mechanisms. In another study, clarified juices led to wines presenting lower content of iron and higher content in copper (Hsia et al., 1975). These transition metals are closely tied to oxidative processes since they act as oxidation catalysts on some polyphenols present in wine (Danilewicz, 2003, 2011). Some authors even proposed that clarified juices could produce white wines that are more stabilized toward oxidation, and that the pale yellowish color of the resulting white wine is more stable (Ribérau-Gayon et al., 1975).

In the context of bottle-aging evolution, oxygen diffusion through the stopper determines wine sensory description (Godden et al., 2001; Skouroumounis et al., 2005; Kwiatkowski et al., 2007; Ugliano, 2013) and could be in certain cases responsible of oxidative deviations and has been shown to be associated to carbonyl off flavors (Bueno et al., 2010) and generating complex chemical modifications in wine matrices (Roullier-Gall et al., 2016).

In this study, we combined a polyphenolic and a macromolecular analysis of bottle-aged Chardonnay white wines with their REDOX sensory scores in order to describe the chemical changes related to oxidative evolution. For that, we work on the impact of must clarification on bottled dry white wine aging evolution through two types of closures: synthetic coextruded stoppers and screw caps. Chardonnay dry white wines from two consecutive vintages (2009 and 2010), elaborated at three different levels of must turbidity, and bottled with two kinds of stoppers, revealed different oxidative evolution trends upon time. We looked here for chemical markers that should be potentially linked to reductive/oxidative perceptions.

## Materials and methods

### Wines

Chardonnay dry white wines were elaborated identically during vintages 2009 and 2010. Chardonnay grapes were hand harvested and pneumatically pressed. Must was protected with a 4 g hL^−1^ SO_2_. Cold racking at 12°C from 12 to 24 h was carried in order to obtain three levels of must clarification, denominated for the present study as “Low,” “Medium,” and “High” corresponding to 300, 600, and 800 NTU, respectively. Musts were directly placed in oak barrels for the alcoholic and malolactic fermentations, and aged for 8 months. Wines were then bottled and 40 mg L^−1^ of SO_2_ was added. Two different closures, including screw caps and synthetic coextruded stoppers, were used to study the further impact of oxygenation during bottling. A total of 12 bottles for each vintage (3 levels of turbidity × 2 types of stopper, in duplicate) were stored in a cellar with constant temperature and humidity and opened in 2015 for chemical and sensory analysis.

### Chemical analysis

#### Chemicals

The water used was purified in ultrapure Millipore Milli-Q (18.2 MΩ cm, Millipore, Merck KGaA, Darmstadt, Germany), methanol, ethanol, acetonitrile, formic acid, tartaric acid and trifluoroacetic acid were purchased from Interchim (Montluçon, France) at the highest grade of purity. A caustic soda solution at 50% (w/w) was supplied by Sigma-Aldrich (St. Louis, MO). Pure standards of gallic acid, protocatechuic acid, hydroxybenzoic acid, hydroxytyrosol, tyrosol, catechin, (2)-epicatechin, dimer B1 and B2, caftaric acid, gentisic acid, caffeic acid, coumaric acid, chlorogenic acid and ferulic acid were obtained from Extrasynthèse (Genay, France).

#### Wine polyphenols

White wine polyphenols were separated and quantified on an Acquity Waters UPLC-DAD-fluorometer. The column was a BEH C18, with a granulometry of 1.7 μm, an internal diameter of 2.1 mm and a length of 150 mm, protected by a guard column packed with the same material. Column temperature was kept constant at 30°C, and samples were held at 8°C. An elution was applied starting isocratic from 100% A (ultrapure water, 0.1% formic acid) from 0 to 6 min and then increasing linearly over 56 min to 100% B (methanol, 0.1% formic acid) where it was held isocratic until 60 min, with a 0.3 mL min^−1^ flow rate. Injection volume was 5 μL. Wine polyphenol concentrations were determined following a calibration made from a polyphenol standards mixture with concentrations ranging from 0.1 to 10 mgL^−1^. For coutaric acid and grape reaction product, concentrations were expressed in mg L^−1^ caftaric acid equivalent. The area of eluted compounds were measured by the Waters Empower software. The chromatographic methodology was improved from a previous study (Coelho et al., 2015). Typical chromatograms are represented in Figure S1. Chemically pure standards of gallic acid, protocatechuic acid, hydroxybenzoic acid, hydroxytyrosol, tyrosol, catechin, (2)-epicatechin, dimer B1 and B2, caftaric acid, gentisic acid, caffeic acid, coumaric acid, chlorogenic acid and ferulic acid were prepared by dilution with water. Gallic acid, protocatechuic acid, hydroxybenzoic acid, hydroxytyrosol, tyrosol, catechin, (2)-epicatechin, dimer B1 and B2, GRP (Grape reaction product), coutaric acid, caftaric acid, gentisic acid, caffeic acid, coumaric acid, chlorogenic acid, ferulic acid and salicylic acid were detected and grouped together into families of phenolic compounds. Caffeic acid, coumaric acid, chlorogenic acid, ferulic acid, caftaric acid and coutaric acid belong to the cinnamic acid family; gallic acid, protocatechuic acid, hydroxybenzoic acid, gentisic acid and salicylic acid belong to the phenolic acid family; catechin, (2)-epicatechin, dimer B1 and B2 belong to the Flavan-(3)-ol family, and hydroxytyrosol and tyrosol to the Tyrosol family.

#### Wine colloids

Wine colloids were separated on a size exclusion chromatography system (VWR LaChromElite®, Radnor, PA) equipped with a Phenomenex Yarra SEC-2000 column with a length of 30 cm, diameter of 7.8 mm, granulometry of 3 μm and pore sizes of 145 Å (Phenomenex, Sartrouville, France). The chromatographic system consists of a L-2130 pump equipped with low-pressure gradient accessory and in-line degasser, a L-2200 autosampler with a sample cooling system, a L-2300 column oven, a L-2455 diode array detector and a L-2485 fluorescence detector (VWR, LaChromElite®, Radnor, PA) The eluting phase was a hydroalcoholic solution of ethanol/water 12/88, (v/v) with 6 g L^−1^ of tartaric acid and a pH adjusted to 3.4, with a caustic soda solution at 50% (w/w). The flow rate was constant at 0.5 mL min^−1^. Fluorescence detection was realized at an emission of 320 nm with an excitation of 280 nm. Injection volume was 20 μL. The system was calibrated with protein standard markers (Sigma Aldrich) to analyse wine colloids with molecular weight ranging from 5 to 300 kDa, and adapted from a recent comparative study (Coelho et al., 2017) using the following standards: Bovine Thyroglobulin (670 kDa), Ferritin (440 kDa), IgG (150 kDa), Ovalbumin (44 kDa), carbonic anhydrase (29 kDa) Ribonucléase (13.7 kDa) and uridin (244 Da). Wine colloids content were determined by integrating the calibrated area below the size exclusion chromatogram, using EZChrom Elite® software. The calibration curve and wine colloids content are shown in Figure S2.

#### Wine proteins

Wine proteins were separated by precipitating 500 μL of white wine with trichloroacetic acid (15%). A total of eight white wines from vintage 2010, coming from this same experimentation, elaborated from low and high must turbidity and bottled with the two kinds of stoppers, were analyzed in triplicate. The precipitated extracts were reduced, alkylated and digested with trypsin. A 2 pM solution of rat serum albumin was used as a digestion quality control. Peptides were separated on a nanoLC/ESI-Trap (Ultimate 3000/Orbitrap ELITE, ThermoScientific) equipped with a LC Thermo Scientific™ Acclaim™ PepMap™ 100 C18 (*L* = 15 cm, *d* = 0,75 cm, granulometry of 3 μm) and optimized from a previous study (Morzel et al., 2012). The mobile phase was made of a mixture of solvent A (ultrapure Milli-Q water 97.9%, acetonitrile 2%, formic acid 0.1%) and solvent B (ultrapure Milli-Q water 19.9%, acetonitrile 80%, formic acid 0.1%). Peptides were eluted in a 120 min gradient mode: 0′: 10% B, 100′: 35% B, 105′: 98% B, 109′: 98% B, 110′: 10% B and 120′: 10% B. Mass data acquisition was made by one scan in the window 400–1,700 m/z, with a resolution of 120,000; followed by 20 scans in MS/MS on the 20 more intense ions from the previous MS scan.

Peptides were matched with wine proteins by using the MASCOT software (www.matrixscience.com) within the NCBI, Tandem and Comet databases. Validation of proteins was carried via Protein and PeptideProphet (tools.proteomecenter.org/TPP.php). Peptides were filtered in order to keep peptides that were found in two of the three technical replicates for one white wine sample. Peptides retention times were aligned following the alignment method from MassChroQ software (Valot et al., 2011). Peptides were quantified with the same software by integrating peak areas on extracted ion chromatograms. A quality control of the nano-LC separation and of the ESI-Trap mass detection was realized on a 0.1 pM BSA standard solution (Table S2). A reproducibility control enabled to isolate 170 peptides and 123 proteins over the eight white wines.

### Sensory analysis

Twenty six oenology students from the University of Burgundy were trained for 2 months on the wine oxidation and reduction aromas. A final selection step based on their sensory performances helped to determine the 11 best tasters. Training and selection protocols were carried according to the ones recently described (Ballester et al., 2018). Sensory analysis took place in a sensory room equipped with individual booths. 2009 samples were assessed in the one session and 2010 samples were analyzed in a second session 24 h later. Samples were served (approximately 30 mL) in standardized black glasses coded by three-digit numbers and assessed monadically according to a Latin square. Each sample was rated using a REDOX sensory score from −5 (strong reductive aroma) to +5 (strong oxidative aroma) being zero the middle point of the scale labeled “neither reduced nor oxidized” (see Ballester et al., 2018 for more details). Participants were asked to rate the redox character of the samples orthonasally (nose only) and afterwards globally (nose and palate) as proposed by Ballester et al. (2018).

### Statistical analysis

Differences in REDOX sensory scores were statistically tested using analysis of variance (ANOVA) to (α = 5%). These data were subjected to three-way ANOVAs with wine closures (synthetic stopper or screw cap) and turbidity (300, 600, and 800 NTU) as within subject factors. The turbidity x closure interaction was also tested. When a significant effect was reached, pairwise comparisons were carried using Newman-Keuls' test (α = 5%). All statistical analyses concerning sensory analysis were carried out using XLStat 2015 (Addinsoft, Paris, France).

The effect of must turbidity on wine polyphenolic and colloidal content and on sensory ratings was analyzed by a one-way ANOVA and a Tukey multiple comparison test taking must turbidity and vintage as factors with a confidence interval of 95%. Prediction of REDOX sensory scores (Y dependent variable) from wine chemical characteristics (predictors) was carried out using partial least-squares regression (PLS) performed with Origin Pro 8.0 software (OriginLab, Northampton, MA).

## Results and discussion

### Phenolic composition

Table 1 gathers values of polyphenol concentrations, expressed according to chemical families. It was obvious that the vintage affects concentrations of phenolic acids, cinnamic acids, flavan-3-ols and GRP but not tyrosol, which could be explained by the fact that this compound is a yeast fermentation marker (Betés-Saura et al., 1996).

**Table 1:** Mean concentrations with standard deviations of white wine polyphenols concentrations, regardless of closures, expressed in mg L^−1^, gathered by chemical families: phenolic acids (gallic acid, protocatechuic acid, hydroxybenzoic acid, hydroxytyrosol, salicylic acid), cinnamic acids (caffeic acid, coumaric acid, ferulic acid, coutaric acid, caftaric acid), flavan-3-ols (catechin and epicatechin), GRP (Grape Reaction Product) and tyrosol.

**Must turbidity**	**Phenolic acids (mg L^−1^)**	**Cinnamic acids (mg L^−1^)**	**Flavan-3-ols (mg L^−1^)**	**GRP (mg L^−1^)**	**Tyrosol (mg L^−1^)**
**VINTAGE 2009**					
Low	4.47 ± 0.07^(A)^	58.61 ± 0.54^(A)^	1.31 ± 0.24^(A)^	3.36 ± 0.07^(A)^	21.97 ± 0.37^(A)^
Medium	4.74 ± 0.08^(A)^	57.52 ± 1.21^(A)^	1.29 ± 0.59^(A)^	3.67 ± 0.05^(B)^	22.80 ± 0.29^(B)^
High	4.41 ± 0.09^(A)^	56.29 ± 0.59^(B)^	1.30 ± 0.28^(A)^	3.65 ± 0.05^(B)^	25.70 ± 0.21^(C)^
**VINTAGE 2010**					
Low	3.75 ± 0.05^(A)^	42.13 ± 0.83^(A)^	0.25 ± 0.12^(A)^	2.69 ± 0.05^(A)^	22.32 ± 0.39^(A)^
Medium	4.20 ± 0.25^(A)^	42.89 ± 0.35^(A)^	0.25 ± 0.14^(A)^	2.90 ± 0.12^(B)^	23.23 ± 0.06^(B)^
High	3.84 ± 0.07^(A)^	44.27 ± 0.70^(B)^	0.39 ± 0.20^(A)^	3.05 ± 0.05^(B)^	23.91 ± 0.23^(C)^

*Letters in superscript present the statistical difference (with α = 5%) obtained between the three levels of turbidity by vintage and for each chemical families*.

For a given vintage, polyphenol concentrations of GRP and tyrosol, appeared to be affected by must racking. Both were significantly more present in bottled white wines (ANOVA with a significance level of 5%) elaborated with less clarified musts (high turbidity), in particular for the 2009 vintage (tyrosol) and the 2010 vintage (GRP). Such differences could be explained by the higher must fermentiscibility (Alexandre et al., 1994; Boivin et al., 1998) or higher must protection against oxidation for unclarified musts (Nagel and Graber, 1988). Interestingly, cinnamic acids were also found statistically different for 2009 and 2010 bottled white wines elaborated from a high must turbidity in a vintage dependent manner. Wines elaborated at high must turbidity presented concentrations of cinnamic acids that decreased in 2009 but increased in 2010 when compared to wines elaborated at low and medium turbidity. By looking in detail among the analyzed polyphenols in Table S1, we found that caftaric acid is the main contributor to this observed trend. In contrast, wine exhibited similar concentrations of phenolic acids and flavan-3-ols, independently of the level of must clarification, meaning that bottle storage did not affect such wine polyphenols. Moreover, it was interesting to notice the relative homogeneity among concentration values for each polyphenol classes, witnessing to a good reproducibility in white winemaking operations. When regarding to the values of flavan-3-ols by taking in account the closures, catechin and epicatechin concentrations were heterogeneous consistently with their reactivity toward oxidation (Arapitsas et al., 2012). Recently we also showed that flavan-3-ols were positively correlated to REDOX sensory scores in white wines (Ballester et al., 2018).

### Macromolecular content

#### Global colloidal content

**Figure 1:**
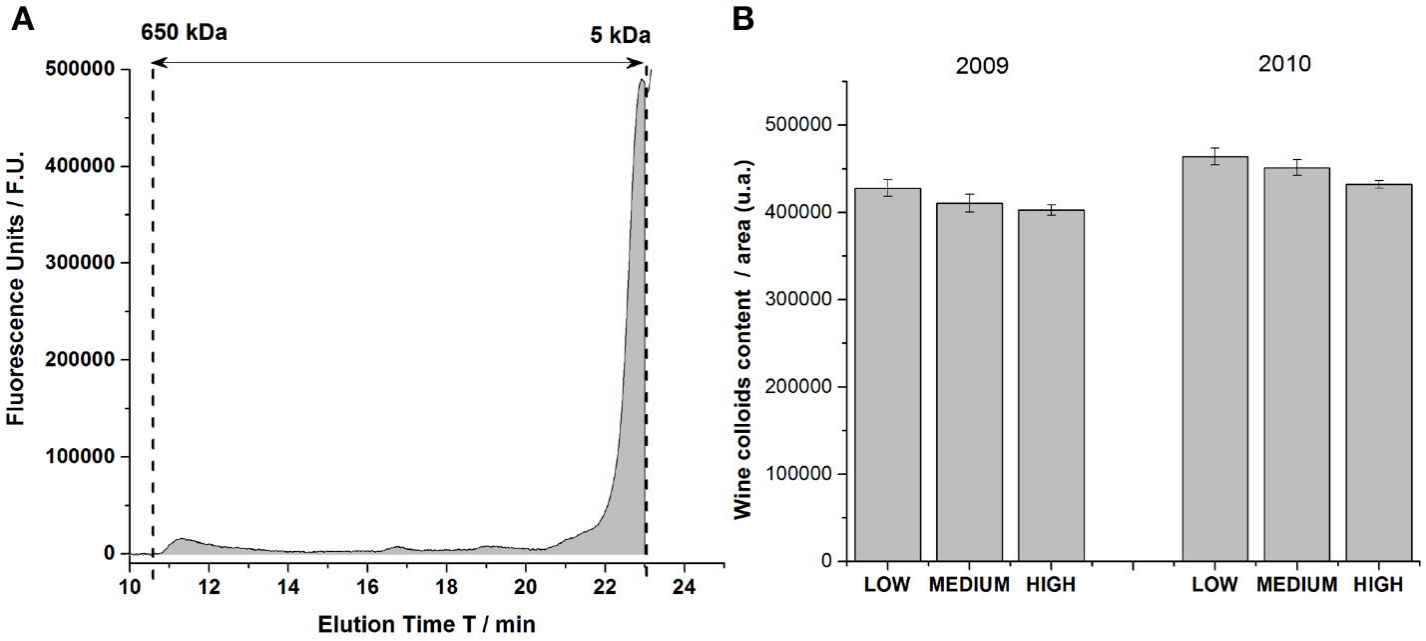
**(A)** Chromatogram of a chardonnay wine in the calibrated domain comprised between 5 kDa and 650 kDa, obtained by size exclusion chromatography. The gray-filled area under the chromatogram represented the total area of wine colloids contained in this wine, and detected by fluorescence. **(B)** Histogram representing the overall colloids content for vintage 2009 and 2010 for the three levels of must turbidity (Low, Medium and High). Each bar of the histogram gathered the medium value of colloids content from 4 bottles (2 types of closure × 2 replicates), with its error bar.

Figure 1A Shows a typical chromatogram of wine colloids in the calibrated area. Under our chromatographic conditions, macromolecules were eluted between 11 and 23 min, which represented molecular weights comprised between 5 and 650 kDa. Most of the detected signal came from relatively low molecular weight compounds ranging from 5 to 30 kDa, that could be easily assimilated to wine oligopeptides and proteins originating from grapes or microorganisms (Coelho et al., 2017). Three other peaks were also present in the chromatogram at 11, 16.4, and 19 min. The eluting peak around 11 min could correspond to high molecular weight mannoproteins coming from yeast autolysates (Charpentier et al., 2004). The total area under the chromatogram in the calibrated domain was represented in Figure 1A.

First, wine colloid contents in vintages 2009 and 2010 were rather similar, in terms of concentration. Anova statistical analyses revealed a vintage effect: vintage 2010 was richer in macromolecular fractions ranging between 5 and 650 kDa. Additionally, bottle closure had very little impact on the concentration of wine colloids as indicated by the small error bars in Figure 1B. In agreement with former studies (Guilloux-Benatier et al., 1995; Boivin et al., 1998), it also appeared, for both vintage, that the level of must clarification modulated the colloidal content in white wines, with higher concentrations in wines from more clarified musts (low turbidity). Even if we could easily suspect a higher production of exocellular and parietal mannoproteins during the fermentative steps for more clarified musts, most of wine colloids presenting low molecular weights from 5 to 40 kDa explained partly the global diminishing of the colloidal content with the increasing level of must clarification, for each vintage. Such results had never been observed so far for analyses run shortly after the fermentation, but were revealed here after 5 and 6 years of bottle aging. This original result, which could be associated with an in-bottle colloidal aging behavior of some wine constituents, further relates to the chemical memory of bottle-aged wines (Roullier-Gall et al., 2017). Such macromolecular compounds represented the main contributors for the white wine chromophoric colloidal matter (Coelho et al., 2017), originating from grape and yeast proteins.

#### Protein content

**Figure 2:**
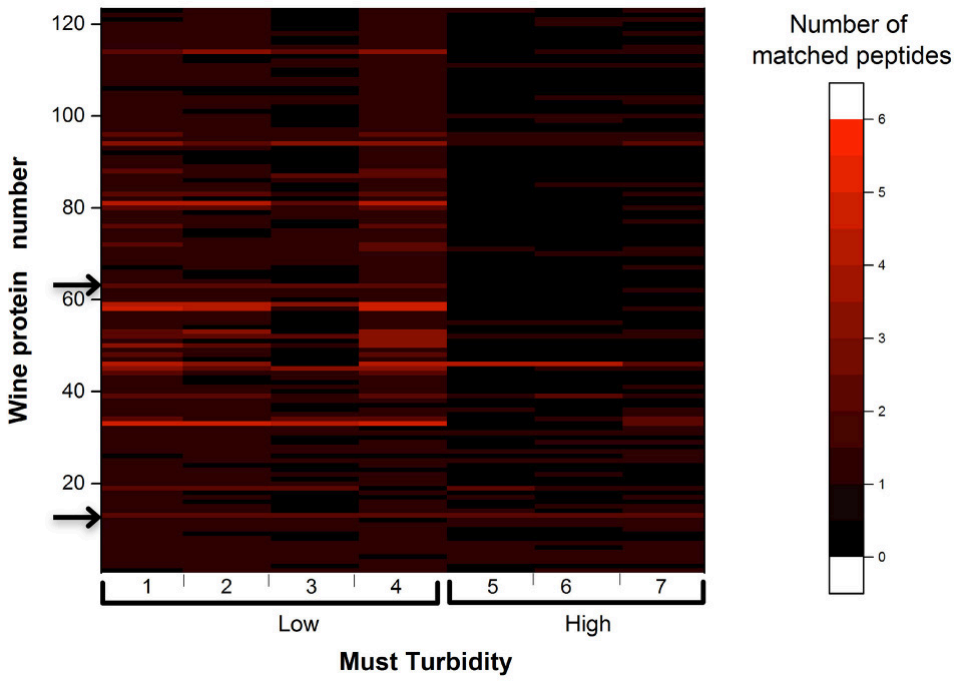
Heat map representing the number of matched peptides among the 123 wine proteins found in Chardonnay white wines elaborated from low turbidity (Low) and high turbidity (High) musts for the vintage 2010. One of the four wines, obtained from a high turbidity must, used in the experimentation has been discarded due to the lowest presence of wine proteins, and peptide retention time alignment problems. Arrows on the left indicate proteins N°14 and 63.

As presented in Figure 2, the proteomic analysis of eight Chardonnay white wines from vintage 2010 revealed that a total of 123 proteins with molecular weights spanning from 8 to 180 kDa, containing between 1 and 5 matched peptides, were significantly representative of the wine proteic content. Figure 2 presented in the ordinates: the number of wine proteins; in the abscissa: the analyzed white wine in function two must turbidities (low and high) used for white wine elaboration for 2010 vintage; and on the z-axis: the number of matched peptides for individual wine proteins. The number of matched peptides in the samples were represented by the intensity of the red color. For example, the wine protein number 14 had been identified as a beta-fructofuranosidase from *Vitis vinifera* grapes (accession number 225466093) and presented two matched peptides SSLAVDDVDQR and TFFCTDLSR, with molecular weight of 60,279,412 and 57,376,632 Da, respectively. These two matched peptides were found for the seven analyzed wines and seemed to be still present after 5 years of bottling, regardless of the level of must clarification.

In contrast, some proteins were only present in wines elaborated from a low must turbidity, like for instance the wine protein number 63, identified as pyruvate decarboxylase isozyme (accession number P06169) and presenting two matched peptides NATFPGVQMK and NPVILADACCSR, with molecular weight of 54,677,923 and 68,832,675 Da, respectively. Such protein, which is known to be involved during the Ehrlich pathway (Hazelwood et al., 2008) appeared to be more present in wines made from clarified musts. Globally, in this experimentation, low turbidity musts led to higher protein contents in resulting wines, in agreement with above SEC results.

### Sensory description of bottle aged white wines

The ANOVA on the 2009 data showed significant effect for closure type (*F* = 4.7, *p* = 0.01) turbidity level (*F* = 5.7, *p* = 0.02) but also a significant closure type x turbidity level (*F* = 3.1, *p* = 0.047). The ANOVA of the 2010 data showed only a very significant effect for closure type (*F* = 20.1, <0.0001).

**Figure 3:**
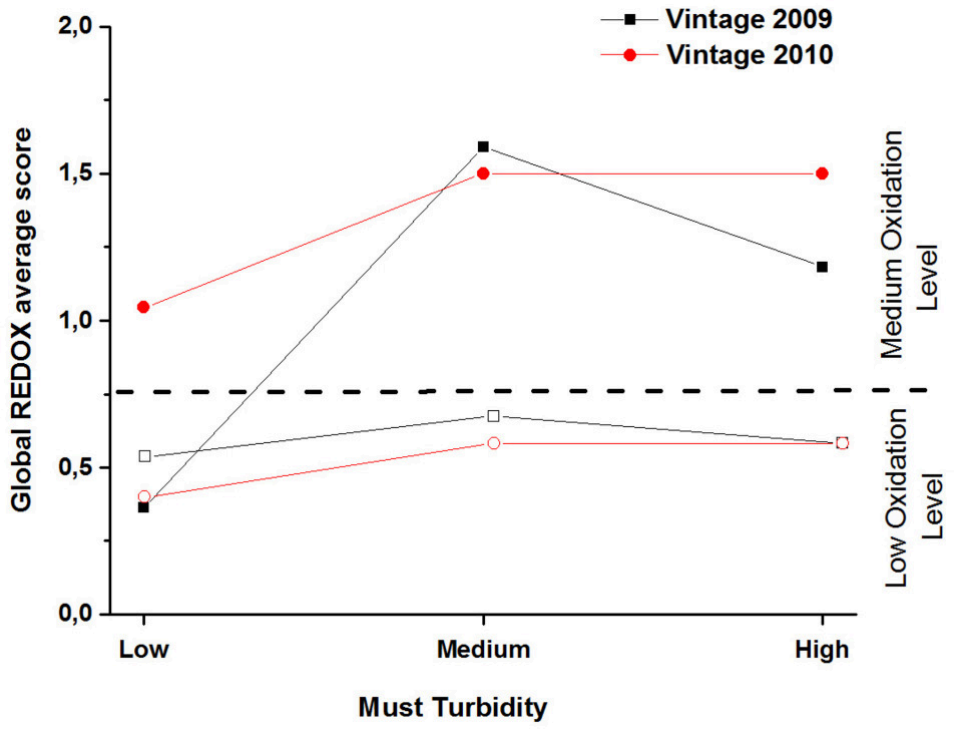
REDOX sensory scores of dry white wines, tasted globally (nose and palate), from vintage 2009 (black) and 2010 (red), plotted against the level of must turbidity and for the two types of bottle closures: synthetic coextruded stopper (filled square and circle symbols) and screw cap (open square and circle symbols).

Figure 3 gathers the average global REDOX sensory scores for 2009 and 2010 samples. This figure clearly shows that screw cap bottled wines presented significantly lower REDOX sensory scores than the ones closed with synthetic coextruded closures, with the exception of the 2009 low turbidity sample (which explains the significant interaction for 2009 data). As previously showed in the literature (Godden et al., 2001; Skouroumounis et al., 2005; Kwiatkowski et al., 2007; Ugliano, 2013), screw caps preserve better from oxidation than synthetic coextruded closures after 5 and 6 years of bottle aging. Based on our results in Figure 3, we defined two oxidation intervals: one from 0.36 (2009/low turbidity/synthetic coextruded closure) to 0.68 (2009/medium turbidity/screw cap) defined as “low oxidation level” and a second one from 1 (2010/low turbidity/synthetic coextruded closure) to 1.6 (2009/medium turbidity/synthetic coextruded closure) defined as “medium oxidation level.”

Concerning the turbidity effect for 2009, according to the post hoc Newman-Keuls comparison, the lower level was significantly less oxidized than the medium level (average REDOX sensory scores 0.45 and 1.14 respectively) but not than the high level (average sensory REDOX score 0.89). High and medium levels were not significantly different.

### Sensory characteristics and correlations with wine chemical composition

**Figure 4:**
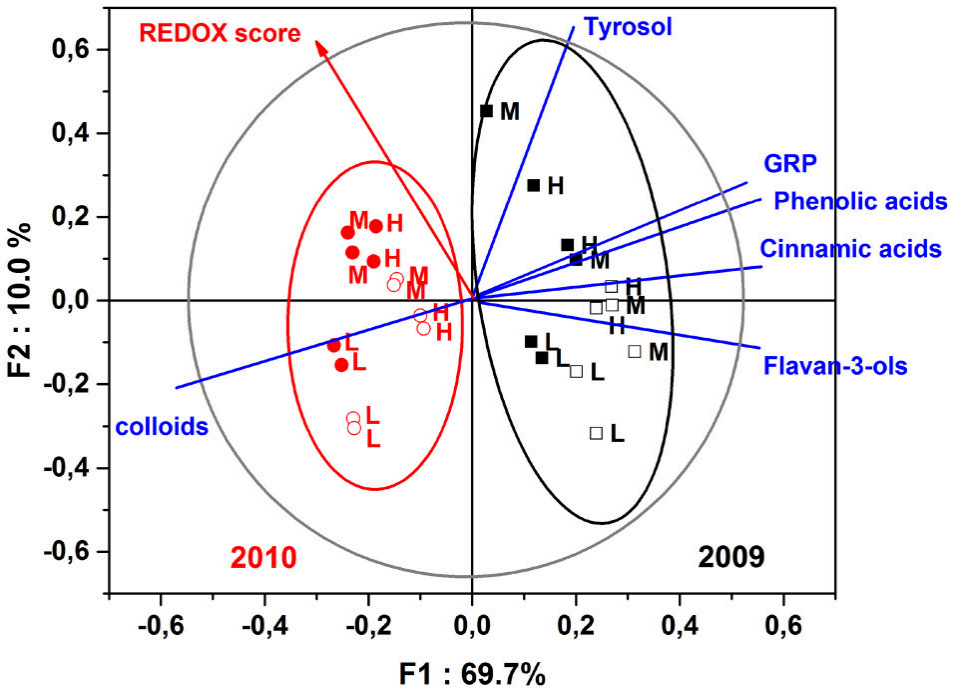
Partial least square—discriminant analysis (PLS-DA) of white wines elaborated from three different must turbidity: low (L), medium (M), and high (H) for vintages 2009 (black) and 2010 (red), and the two types of bottle closures: synthetic coextruded stopper (filled square and circle symbols) and screw cap (open square and circle symbols). Scores and loadings plots of the PLS-DA analysis of the REDOX sensory scores averaged for each sample as a function of the polyphenolic and colloidal contents.

The next step of this experimentation was to establish correlations existing between sensory attributes and the chemical composition. To that purpose, Figure 4 illustrates the correlations existing between REDOX sensory scores and polyphenolic and colloidal content of tasted dry white wines, for both 2009 and 2010 vintages and the two bottle closures.

The first two factors of the PLS-DA explained 79.7% of the variance, where the first factor F1 clearly distinguished vintages in terms of chemical composition and REDOX sensory score. Vintage 2009 was: richer in polyphenols, particularly more cinnamic acids; poorer in colloidal matter; and presented lower values of oxidation ratings compared to vintage 2010. The second factor F2 discriminated nicely the must turbidity parameter for both vintages and particularly wines made from low turbidity musts, which were correlated to high contents of wine colloidal matter and low REDOX sensory score. The PLS-DA model also took in account our closure effect observation in terms of oxidative perception, for a given vintage and level of turbidity, where non oxidized screw capped wines presented highest F1 scores and lowest F2 scores compared to the oxidized wines that were closed synthetically.

We therefore focused our study in the vintage 2010 which presented the highest amount of colloidal matter and particularly wine proteins for the two bottle closures (screw cap and synthetic coextruded stoppers) in order to cover a large window of wine REDOX sensory scores. The aim was to statistically investigate the possible associations existing between wine proteins content and the level of oxidation reached by each white wines. In that context, a partial least square analysis was carried in order to discriminate among the 123 wine proteins, those that best discriminated white wines in function of their REDOX sensory scores. As shown in Table 2, a list of 10 proteins were proposed as being potential markers for wines sensorially clustered in the medium oxidation level, presenting higher REDOX sensory scores compared to those clustered in the low oxidation level.

**Table 2:** Ten proteins that best discriminated white wines from the vintage 2010 according to their low vs. medium oxidation levels, with indication of their average number of matched peptides.

**Accession number**	**Protein name**	**Mean of matched peptides on wines with**	
		**Low oxidation**	**Medium oxidation**
		**Level**	**Level**
147774756	Hypothetical protein VITISV_024840	0.33	**1**
296083086	Unnamed protein product, partial	**1**	0.5
359476759	Basic Leucine zipper and W2 domain-containing protein 2	**1**	0.25
359483753	Low-temperature-induced cysteine proteinase-like	**2**	1
675082338	Chain B, Structure of haze forming proteins in white wine, Vitis vinifera thaumatin-like proteins	**0.67**	0
731398090	Putative pentatricopeptide repeat-containing protein	**0.67**	0
731413051	Actin-1	1	**1.75**
731419561	Structural maintenance of chromosomes protein 4 isoform X2	**1**	0.5
731421658	Uncharacterized protein LOC100263595	**0.67**	0
P02994	Elongation Factor 1-alpha	**1**	0.5

*Bold values represent the highest mean values of matched peptides between low and medium oxidation level*.

Eight of them presented on average more peptides for low REDOX wines compared to high REDOX wines. The low-temperature-induced-proteinase-like protein was the most representative for low REDOX white wines, containing in average two matched peptides. Four others proteins were significantly more present in low REDOX wines, but with only one matched peptide: unnamed protein product, basic leucine zipper and W2 domain-containing protein, structural maintenance of chromosomes protein 4 isoform X2 and elongation factor 1-alpha.

Two of them: actin-1 and hypothetical protein VITISV_024840 presented on average more peptides for high REDOX wines compared to low REDOX wines: 1.75 and 1, respectively. This result suggests that these two grape proteins could be involved in oxidation mechanisms. A study carried on actin from *Saccharomyces cerevisiae* showed its functional role in the regulation of the oxidative stress in the yeast metabolism (Farah and Amberg, 2007) where disulfides bonds formed between cysteine moieties of actins resulted from an elevated activity of reactive oxygenated species, formed during oxidation. Here, among the matched peptides from the two grape proteins, only methionine and not cysteine, was present. This sulfur-containing amino could be responsible for the preservation of sulfur-containing proteins in oxidized wines via the formation of methionine sulfoxides (Levine et al., 1996). Such compounds have been shown to be efficient against oxidant scavengers that could be more present in wines with an oxidative history. Actin and hypothetical protein VITISV_024840 were good protein candidates to elucidate the oxidative stability of white wines, and to better assess oxidative deviations.

## Conclusions

We reported here the first combined molecular/macromolecular characterization of bottle aged white wines and its correlation with their oxidative state. To that purpose, wines from the 2009 and 2010 vintages, made from the same must but with three levels of clarification, were analyzed for their polyphenolic and chromophoric macromolecular contents. If polyphenol contents appeared to be mostly driven by the vintage, proteins, contributing mostly to the colloidal fraction studied, were directly correlated to the level of clarification, even after several years of bottle aging, and regardless of the vintage. REDOX sensory scores ranging from −5 to +5, made by a trained sensory panel, further enabled to distinguish wines according to their oxidative perception. The type of bottle closure thus appeared to be responsible for the most significant REDOX sensory scores differences, with screw caps leading to low REDOX sensory scores and thus less oxidized wines, while synthetic coextruded closures led to more oxidized ones. A quantitative proteomic analysis of wines from the 2010 vintage possible correlations existing between levels of must clarification and the oxidative perception of white wines. Two sulfur-containing grape proteins: actin and hypothetical protein VITISV_024840 were thus shown to be potential markers of oxidation evolution. These two grape proteins could be in the future quantitatively analyzed to validate their involvement in oxidation deviations in white wines.

## Author contributions

CC and JB conceived the study. CC analyzed the data and wrote the manuscript. CC, LN, MM, and JB performed the experiments. PJ and JB analyzed the sensory results. MN and RG took part to scientific discussions. All authors contributed to the final form of the manuscript.

### Conflict of interest statement

The authors declare that the research was conducted in the absence of any commercial or financial relationships that could be construed as a potential conflict of interest.

## References

[B1] Alexandre H. Nguyen Van Long T. Feuillat M. Charpentier C. (1994). Contribution à l'étude des bourbes: influence sur la fermentescibilité des moûts. Rev. Oenol. 145, 11–20.

[B2] Ancín C. Ayestarán B. Corroza M. Garrido J. González A. (1996). Influence of prefermentation clarification on the higher alcohol contents of wines. Food Chem. 55, 241–249. 10.1016/0308-8146(95)00125-5

[B3] Arapitsas P. Scholz M. Vrhovsek U. Di Blasi S. Biondi Bartolini A. Masuero D.. (2012). A metabolomic approach to the study of wine micro-oxygenation. PLoS ONE 7:e37783. 10.1371/journal.pone.0037783 22662221 PMC3360592

[B4] Ayestaran B. Guadalupe Z. Leon D. (2004). Quantification of major grape polysaccharides (*Tempranillo* v.) released by maceration enzymes during the fermentation process. Anal. Chim. Acta 513, 29–39. 10.1016/j.aca.2003.12.012

[B5] Ayestaran B. M. Ancin M. C. Garcia A. M. Gonzalez A. Garrido J. J. (1995). Influence of prefermentation clarification on nitrogenous contents of musts and wines. J. Agric. Food Chem. 43, 476–482. 10.1021/jf00050a041

[B6] Ballester J. Magne M. Julien P. Noret L. Nikolantonaki M. Coelho C. (2018). Sensory impact of polyphenolic composition on the oxidative notes of chardonnay wines. Beverages 4:19 10.3390/beverages4010019

[B7] Betés-Saura C. Andrés-Lacueva C. Lamuela-Raventós R. M. (1996). Phenolics in White free run juices and wines from penedès by high-performance liquid chromatography: changes during vinification. J. Agric. Food Chem. 44, 3040–3046. 10.1021/jf9601628

[B8] Boivin S. Feuillat M. Alexandre H. Charpentier C. (1998). Effect of Must Turbidity on Cell Wall Porosity and Macromolecule Excretion of *Saccharomyces cerevisiae* Cultivated on Grape Juice. Am. J. Enol. Vitic. 49, 325–332.

[B9] Bueno M. Culleré L. Cacho J. Ferreira V. (2010). Chemical and sensory characterization of oxidative behavior in different wines. Food Res. Int. 43, 1423–1428. 10.1016/j.foodres.2010.04.003

[B10] Casalta E. Cervi M.-F. Sablayrolles J.-M. Salmon J.-M. (2012). Effet combiné des niveaux d'azote assimilable et de bourbes: nouveau paramétre á prendre en compte pour la maîtrise de la fermentation alcoolique. Revue Française d'Oenologie 255, 9–15.

[B11] Charpentier C. Dos Santos A. M. Feuillat M. (2004). Release of macromolecules by *Saccharomyces cerevisiae* during aging of French flor sherry wine “Vin jaune”. Int. J. Food Microbiol. 96, 253–262. 10.1016/j.ijfoodmicro.2004.03.019 15454315

[B12] Coelho C. Aron A. Roullier-Gall C. Gonsior M. Schmitt-Kopplin P. Gougeon R. D. (2015). Fluorescence fingerprinting of bottled white wines can reveal memories related to sulfur dioxide treatments of the must. Anal. Chem. 87, 8132–8137. 10.1021/acs.analchem.5b00388 26190639

[B13] Coelho C. Parot J. Gonsior M. Nikolantonaki M. Schmitt-Kopplin P. Parlanti E.. (2017). Asymmetrical flow field-flow fractionation of white wine chromophoric colloidal matter. Anal. Bioanal. Chem. 409, 2757–2766. 10.1007/s00216-017-0221-1 28180990

[B14] Danilewicz J. C. (2003). Review of reaction mechanisms of oxygen and proposed intermediate reduction products in wine: central role of iron and copper. Am. J. Enol. Vitic. 54, 73–85. Available online at: http://www.ajevonline.org/content/54/2/73.abstract

[B15] Danilewicz J. C. (2011). Mechanism of autoxidation of polyphenols and participation of sulfite in wine: key role of iron. Am. J. Enol. Vitic. 62, 319–328. 10.5344/ajev.2011.10105

[B16] Farah M. E. Amberg D. C. (2007). Conserved actin cysteine residues are oxidative stress sensors that can regulate cell death in yeast. Mol. Biol. Cell 18, 1359–1365. 10.1091/mbc.E06-08-0718 17287397 PMC1838977

[B17] Ferreira B. Hory C. Bard M. H. Taisant C. Olsson A. Le Fur Y. (1995). Effects of skin contact and settling on the level of the C18:2, C18:3 fatty acids and C6 compounds in burgundy chardonnay musts and wines. Food Qual. Prefer. 6, 35–41. 10.1016/0950-3293(94)P4210-W

[B18] Gawel R. Day M. Van Sluyter S. C. Holt H. Waters E. J. Smith P. A. (2014). White wine taste and mouthfeel as affected by juice extraction and processing. J. Agric. Food Chem. 62, 10008–10014. 10.1021/jf503082v 25248855

[B19] Gerbaux V. Meurgues O. (1996). Influence du sulfitage et du débourbage des moûts sur l'élaboration et la qualité des vins de chardonnay. Rev. Oenol. 78, 15–18.

[B20] Godden P. Francis L. Field J. Gishen M. Coulter A. Valente P. (2001). Wine bottle closures: physical characteristics and effect on composition and sensory properties of a Semillon wine 1. Performance up to 20 months post-bottling. Austr. J. Grape Wine Res. 7, 64-105. 10.1111/j.1755-0238.2001.tb00196.x

[B21] Groat M. Ough C. S. (1978). Effects of insoluble solids added to clarified musts on fermentation rate, wine composition, and wine quality. Am. J. Enol. Vitic. 29, 112–119.

[B22] Guilloux-Benatier M. Feuillat M. (1993). Incidence de la clarification des moûts de raisin sur les fermentescibilités alcoolique et malolactique. J. Inter. Sci. Vig.Vin 4, 299–311.

[B23] Guilloux-Benatier M. Guerreau J. Feuillat M. (1995). Influence of Initial Colloid Content on Yeast Macromolecule Production and on the Metabolism of Wine Microorganisms. Am. J. Enol. Viticult. 46, 486–492.

[B24] Hazelwood L. A. Daran J.-M. van Maris A. J. A. Pronk J. T. Dickinson J. R. (2008). The ehrlich pathway for fusel alcohol production: a century of research on *Saccharomyces cerevisiae* metabolism. Appl. Environ. Microbiol. 74, 2259–2266. 10.1128/AEM.02625-07 18281432 PMC2293160

[B25] Hsia C. L. Planck R. W. Nagel C. W. (1975). Influence of must processing on iron and copper contents of experimental wines. Am. J. Enol. Vitic. 26, 57–61.

[B26] Kwiatkowski M. J. Skouroumounis G. K. Lattey K. A. Waters E. J. (2007). The impact of closures, including screw cap with three different headspace volumes, on the composition, colour and sensory properties of a Cabernet Sauvignon wine during two years' storage. Aust. J. Grape Wine Res. 13, 81–94. 10.1111/j.1755-0238.2007.tb00238.x

[B27] Lavigne V. (1996). Recherche sur les Composés Volatils Soufrés Formés par la Levure au Cours de la Vinification et de L'élevage des vins Blancs Secs. Thèse doctorat, Université de Bordeaux II.

[B28] Lavigne V. Dubourdieu D. (1997). Recherche sur les composés soufrés formés par la levure au cours de la vinification et de l'élevage des vins blancs secs. Rev. Oenol. 85, 23–30.

[B29] Levine R. L. Mosoni L. Berlett B. S. Stadtman E. R. (1996). Methionine residues as endogenous antioxidants in proteins. Proc. Natl. Acad. Sci. U.S.A. 93, 15036–15040. 8986759 10.1073/pnas.93.26.15036 PMC26351

[B30] Liu J.-W. R. Gallander J. F. Wilker K. L. (1987). Effect of juice clarification on the composition and quality of eastern US table wines. Am. J. Enol. Vitic. 38, 147–150.

[B31] Morzel M. Jeannin A. Lucchi G. Truntzer C. Pecqueur D. Nicklaus S.. (2012). Human infant saliva peptidome is modified with age and diet transition. J. Proteomics 75, 3665–3673. 10.1016/j.jprot.2012.04.028 22575268

[B32] Nagel C. W. Graber W. R. (1988). Effect of must oxidation on quality of white wines. Am. J. Enol. Vitic. 39, 1–4.

[B33] Ollivier C. Stonestreet T. Larue F. Dubourdieu D. (1987). Incidence de la composition colloïdale des moûts blancs sur leur fermentescibilité. Connaissance de la Vigne et du Vin 21, 59-70. 10.20870/oeno-one.1987.21.1.1272

[B34] Ribérau-Gayon J. Lafon-Lafourcade S. Bertrand A. (1975). Le débourbage des mouts de vendange blanche. Connaiss. Vigne Vin 9, 117–139. 10.20870/oeno-one.1975.9.2.1807

[B35] Riou V. Vernhet A. Doco T. Moutounet M. (2002). Aggregation of grape seed tannins in model wine—effect of wine polysaccharides. Food Hydrocoll. 16, 17–23. 10.1016/S0268-005X(01)00034-0

[B36] Roullier-Gall C. Hemmler D. Gonsior M. Li Y. Nikolantonaki M. Aron A.. (2017). Sulfites and the wine metabolome. Food Chem. 237, 106–113. 10.1016/j.foodchem.2017.05.039 28763951

[B37] Roullier-Gall C. Witting M. Moritz F. Gil R. B. Goffette D. Valade M.. (2016). Natural oxygenation of Champagne wine during ageing on lees: a metabolomics picture of hormesis. Food Chem. 203, 207–215. 10.1016/j.foodchem.2016.02.043 26948607

[B38] Skouroumounis G. K. Kwiatkowski M. J. Francis I. L. Oakey H. Capone D. L. Peng Z. (2005). The influence of ascorbic acid on the composition, colour and flavour properties of a Riesling and a wooded Chardonnay wine during five years' storage. Aust. J. Grape Wine Res. 11, 355–368. 10.1111/j.1755-0238.2005.tb00035.x

[B39] Ugliano M. (2013). Oxygen Contribution to Wine Aroma Evolution during Bottle Aging. J. Agric. Food Chem. 61, 6125–6136. 10.1021/jf400810v 23725213

[B40] Valot B. Langella O. Nano E. Zivy M. (2011). MassChroQ: a versatile tool for mass spectrometry quantification. Proteomics 11, 3572–3577. 10.1002/pmic.201100120 21751374

[B41] Vidal S. Francis L. Williams P. Kwiatkowski M. Gawel R. Cheynier V. (2004). The mouth-feel properties of polysaccharides and anthocyanins in a wine like medium. Food Chem. 85, 519–525. 10.1016/S0308-8146(03)00084-0

[B42] Williams J. T. Ough C. S. Berg H. W. (1978). White wine composition and quality as influenced by method of must clarification. Am. J. Enol. Vitic. 29, 92–96.

