# Supplementary material for: Molecular and Macromolecular Changes in Bottle-Aged White Wines Reflect Oxidative Evolution–Impact of Must Clarification and Bottle Closure
Source: Front Chem. 2018 Apr 6;6:95. doi: 10.3389/fchem.2018.00095 (PMC5897750; doi:10.3389/fchem.2018.00095)

**Supporting Information**

Molecular and macromolecular changes in bottle-aged white wines reflect oxidative evolution – Impact of must clarification and bottle closure

Christian COELHO^1,*^, Perrine JULIEN^2^, Maria NIKOLANTONAKI^1^, Laurence NORET^1^, Mathilde MAGNE^2^, Jordi BALLESTER^2^, Régis D. GOUGEON^1^

^1^UMR A 02.102 PAM laboratoire PAPC AgroSup Dijon, Université de Bourgogne, Institut Universitaire de la vigne et du vin Jules Guyot, rue Claude Ladrey, BP 27877, 21078 Dijon Cedex, France.

^2^UMR UB/INRA/CNRS Centre des Sciences du Goût et de l’Alimentation, Equipe CEP (Culture, Expertise et Perception), Dijon, France.

Table S.I.1. Polyphenolic concentrations, expressed in mg.L^-1^, of all opened white wines taking into account the vintage effect. Each value represents the mean value of four bottled wines with two different closures (screw caps and synthetic coextruded caps)

|  | **Vintage 2009** | | | **Vintage 2010** | | |
| --- | --- | --- | --- | --- | --- | --- |
| Turbidity / NTU | **300** | **600** | **900** | **300** | **600** | **900** |
| Gallic acid | 1.56 ± 0.03 | 1.38 ± 0.04 | 1.02 ± 0.01 | 1.02 ± 0.02 | 1.07 ± 0.02 | 0.83 ± 0.01 |
| Protocatechuic acid | 0.76 ± 0.03 | 0.80 ± 0.04 | 0.74 ± 0.05 | 0.35 ± 0.01 | 0.40 ± 0.03 | 0.39 ± 0.04 |
| Hydroxytryrosol | 1.87 ± 0.05 | 2.30 ± 0.01 | 2.41 ± 0.03 | 2.24 ± 0.05 | 2.58 ± 0.29 | 2.45 ± 0.08 |
| Hydroxybenzoic acid | 0.23 ± 0.01 | 0.26 ± 0.02 | 0.24 ± 0.02 | 0.14 ± 0.01 | 0.15 ± 0.01 | 0.17 ± 0.00 |
| Tyrosol | 21.97 ± 0.37 | 22.80 ± 0.29 | 25.70 ± 0.21 | 22.33 ± 0.39 | 23.23 ± 0.06 | 23.91 ± 0.23 |
| (+) catechine | 1.27 ± 0.18 | 1.20 ± 0.50 | 1.24 ± 0.22 | 0.25 ± 0.12 | 0.25 ± 0.14 | 0.39 ± 0.20 |
| (-) epicatechine | 0.15 ± 0.08 | 0.18 ± 0.11 | 0.13 ± 0.07 | ND | ND | ND |
| Salycilic acid | 0.18 ± 0.09 | ND^a^ | ND^a^ | ND^a^ | ND^a^ | ND^a^ |
| Caftaric acid | 45.81 ± 0.22 | 44.76 ± 0.21 | 43.63 ± 0.30 | 34.67 ± 0.45 | 35.81 ± 0.55 | 36.75 ± 0.28 |
| GRP (Grape Reaction Product) ^b^ | 3.36 ± 0.07 | 3.67 ± 0.04 | 3.65 ± 0.05 | 2.69 ± 0.05 | 2.91 ± 0.12 | 3.06 ± 0.05 |
| Coutaric acid ^b^ | 8.26 ± 0.28 | 8.15 ± 0.75 | 7.85 ± 0.28 | 4.43 ± 0.29 | 4.55 ± 0.27 | 4.67 ± 0.32 |
| Caffeic acid | 3.10 ± 0.03 | 3.15 ± 0.19 | 3.30 ± 0.10 | 2.22 ± 0.03 | 1.84 ± 0.10 | 2.18 ± 0.07 |
| Coumaric acid | 1.21 ± 0.02 | 1.21 ± 0.09 | 1.28 ± 0.06 | 0.69 ± 0.09 | 0.58 ± 0.11 | 0.54 ± 0.07 |
| Ferulic acid | 0.23 ± 0.00 | 0.24 ± 0.01 | 0.24 ± 0.01 | 0.12 ± 0.01 | 0.10 ± 0.01 | 0.12 ± 0.01 |

ND^a^: Not Detected,

^b^ : expressed in mg L^-1^ caftaric acid equivalent

Figure S.I.2. Calibration curve of SEC between 5 and 250 kDa (A) and Overall colloids content in the calibrated area, expressed in u.a., of all opened white wines taking into account the closure and the vintage effect (B)

A)


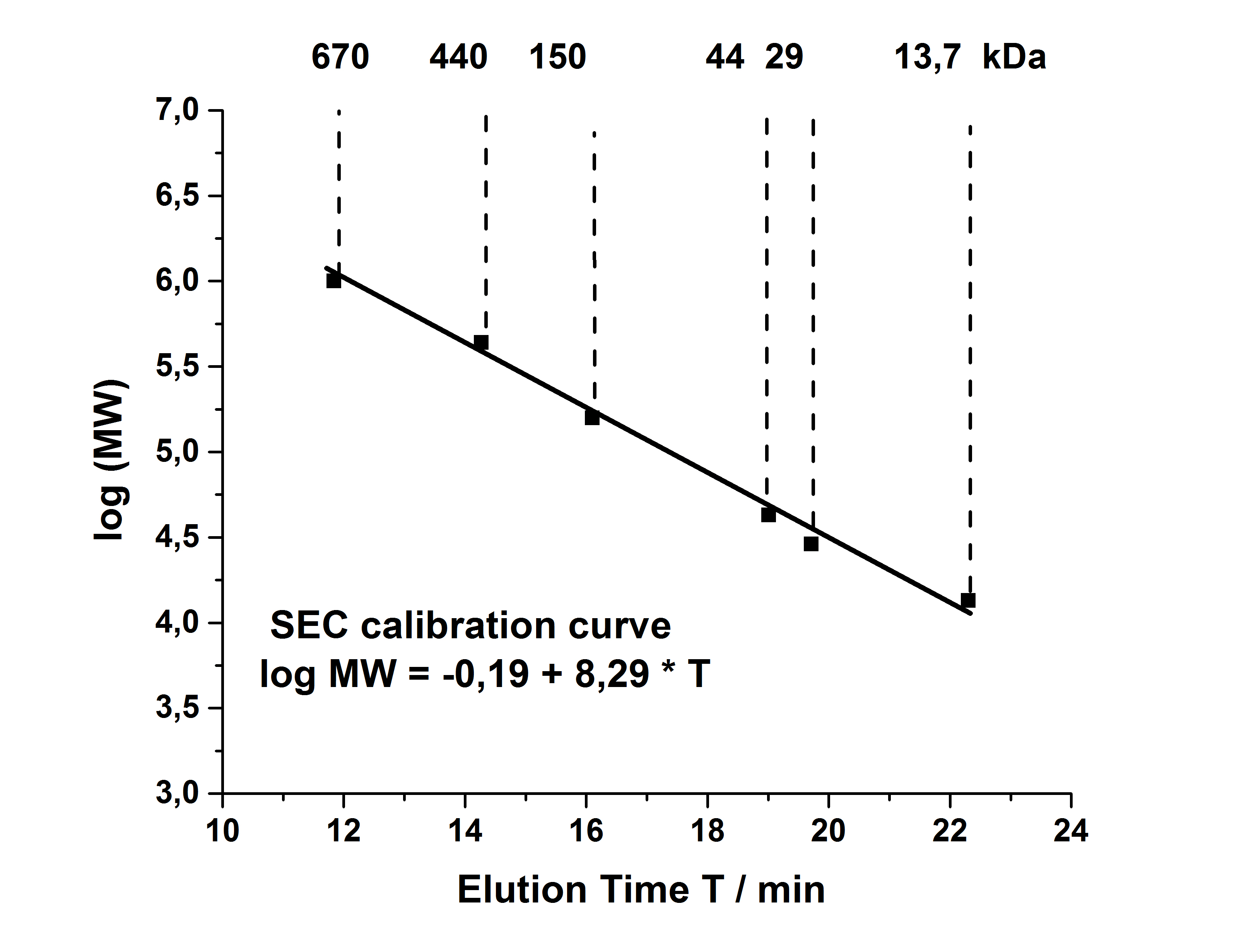


B)

|  | **Vintage 2009** | | | **Vintage 2010** | | |
| --- | --- | --- | --- | --- | --- | --- |
| **Turbidity / NTU** | **300** | **600** | **900** | **300** | **600** | **900** |
| Coextruded synthetic closure | 421180  ± 702 | 403511  ± 519 | 398805  ± 795 | 470889  ± 764 | 445080  ± 597 | 429193  ± 607 |
| Screw cap | 434736  ± 698 | 417843  ± 579 | 407115  ± 782 | 457434  ± 653 | 457797  ± 585 | 435450  ± 693 |
| Both closures (presented in Figure 1 b) | 427962  ± 10081 | 410677  ± 10533 | 402960  ± 6276 | 464161  ± 10013 | 451439  ± 9489 | 432321  ± 5025 |

Table S.I.3. Quality control of nano LC/ESI-Trap realized on a 0.1 pM BSA standard solution representing a coverage of 69% of the intact protein with 45 peptides recovered with a tolerance error of 2 ppm.


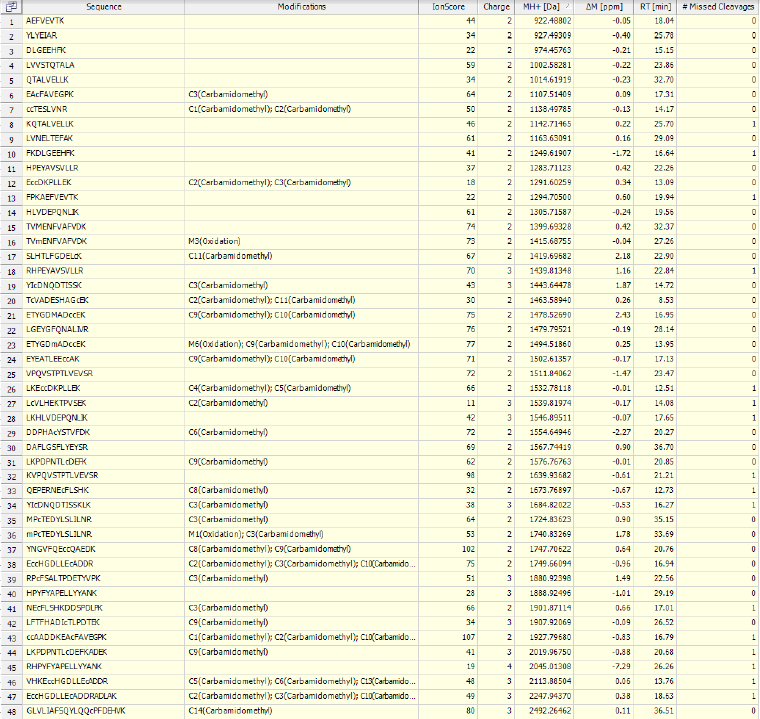

Supplement: Supplementary file 1 [file DataSheet1.docx]
